# Editorial: Zoonotic diseases: epidemiology, multi-omics, and host-pathogen interactions

**DOI:** 10.3389/fmicb.2024.1454659

**Published:** 2024-10-04

**Authors:** Lei Deng, Hong Yin, Kevin S. W. Tan, Anastasios D. Tsaousis

**Affiliations:** ^1^Infectious Disease and Microbiome Program, Broad Institute of MIT and Harvard, Cambridge, MA, United States; ^2^Center for Computational and Integrative Biology, Massachusetts General Hospital, Boston, MA, United States; ^3^African Swine Fever Regional Laboratory, China (Lanzhou) and State Key Laboratory of Veterinary Etiological Biology and Key Laboratory of Veterinary Parasitology of Gansu Province, Lanzhou Veterinary Research Institute, Chinese Academy of Agricultural Sciences, Lanzhou, China; ^4^Laboratory of Molecular and Cellular Parasitology, Healthy Longevity Translational Research Programme and Department of Microbiology and Immunology, Yong Loo Lin School of Medicine, National University of Singapore, Singapore, Singapore; ^5^Laboratory of Molecular and Evolutionary Parasitology, School of Biosciences, University of Kent, Canterbury, United Kingdom

**Keywords:** zoonotic diseases, epidemiology, multi-omics, host-pathogen interactions, global health

Zoonotic diseases, which can be transmitted between humans and animals, pose a significant risk to global health. These infections, caused by various microorganisms such as viruses, bacteria, parasites, and fungi, often lead to severe and life-threatening illnesses. However, some infected individuals may remain asymptomatic. Remarkably, almost 60% of human diseases are zoonotic, and at least 75% of emerging infectious diseases are of zoonotic origin.

Despite their impact, many zoonotic diseases are neglected. This Research Topic aims to explore the pathogenesis, molecular evolution, transmission dynamics, and host interaction mechanisms of zoonotic pathogens, providing a platform for innovative research focused on preventing and controlling zoonotic diseases. Within this topic, 17 research articles and five reviews have been published that advanced our knowledge on zoonotic diseases.

Huang et al. investigated tick-borne viruses, particularly the Jingmen tick virus (JMTV), discovering a novel strain, the Sichuan tick virus, with implications for cross-species transmission and co-infection in multiple tick hosts. Through high-throughput sequencing, they identified whole virus genomes from four tick samples, revealing a novel JMTV-like virus with signals of reassortment with other JMTV strains, suggesting a complex interplay of viral evolution in ticks. Shah et al. reviewed several critical tick-borne viruses, such as Bourbon virus (BRBV), Dhori virus (DHOV), Powassan virus (POWV), Omsk hemorrhagic fever virus (OHFV), Colorado tick fever virus (CTFV), Crimean-Congo hemorrhagic fever virus (CCHFV), Heartland virus (HRTV), and Kyasanur forest disease virus (KFDV). They highlighted these viruses' eco-epidemiology, pathogenesis, and clinical manifestations, emphasizing the need for targeted preventive measures. Majid et al. identified *Rickettsia* pathogens in various tick species in Pakistan, highlighting the need for comprehensive surveillance to assess potential health risks to humans and livestock. Their study revealed the presence of *Rickettsia* in ticks from domestic and wild hosts, underscoring the importance of monitoring tick populations to prevent zoonotic outbreaks.

Ali et al. conducted molecular screening of *Coxiella* spp. in various ticks from different hosts in Pakistan, identifying several new species and highlighting the need for proper surveillance to mitigate health risks. They confirmed the presence of *Coxiella burnetii* and *Coxiella* endosymbionts in multiple tick species, providing crucial insights into the ecology of these pathogens. Wu S. et al. investigated the prevalence and genetic diversity of piroplasmosis in pet dogs and cats in Guiyang. The study confirmed the presence of *Theileria uilenbergi* and *Theileria luwenshuni* in both cats and dogs, highlighting the importance of surveillance in pet populations to control the spread of these pathogens. They found a significant prevalence of these parasites, indicating a potential public health risk. Zhang X. et al. identified several tick-borne pathogens, including *Rickettsia, Anaplasma*, and *Ehrlichia* species, in ticks collected from domestic cattle and goats, emphasizing the need for comprehensive surveillance to mitigate the risk to human and animal health. Their findings provided a detailed genetic analysis of these pathogens, revealing the diversity and prevalence of tick-borne diseases in the region. Xu et al. identified a new Dabieshan tick virus strain, highlighting the need for expanded surveillance to understand its transmission and pathogenicity. Their study discovered the virus in multiple tick species, suggesting a widespread distribution and potential health impact. Jamil et al. investigated the prevalence of tick-borne pathogens (TBPs) in Pakistan. They collected 213 ticks from various animals, identifying five species: *Hyalomma anatolicum, Rhipicephalus microplus, Hyalomma scupense, Rhipicephalus turanicus*, and *Rhipicephalus sanguineus*. The study underscores a broader range of TBPs in Pakistan, highlighting the need for improved control measures to protect livestock and public health.

Fernandes et al. applied CRISPR interference (CRISPRi) to study the pathogenesis and virulence factors of Leptospira, providing insights into leptospiral biology and potential vaccine targets. They used CRISPRi to silence key leptospiral proteins, demonstrating the role of these proteins in bacterial virulence and host interactions. Meng W. et al. focused on *Corynebacterium pseudotuberculosis* strains isolated from alpacas, exploring their antibiotic resistance and genetic properties to understand their risk to livestock. Their study included antibiotic susceptibility tests and genome sequencing, revealing multiple antibiotic resistance genes and virulence factors.

Špičić et al. identified a novel *Mycobacterium* species in mollusks, contributing to the understanding of mycobacterial infections in marine environments. They used genomic and phenotypic analyses to characterize the new species, proposing *Mycobacterium pinniadriaticum* sp. nov. as the new species name.

The research by Wan et al. explores the role of the CpxAR system in *Actinobacillus pleuropneumoniae* under high potassium (K+) stress, which hinders bacterial growth. They found that CpxAR is crucial for cell division in these conditions by upregulating the cell division genes ftsE and ftsX. qRT-PCR showed positive regulation of these genes, and EMSA demonstrated CpxR-P binding to the ftsE promoter. This study is the first to detail the mechanism by which CpxAR confers high-K+ tolerance in *A. pleuropneumoniae*. Wu Z. et al. investigated the prevalence of *Klebsiella pneumoniae* in pigs. They infected mice and pigs with a human-derived *K. pneumoniae* strain and developed an indirect ELISA using KHE protein and a nested PCR method for detection. The ELISA was optimized, showing high sensitivity and specificity, with an infection rate of 27.28% (ELISA) and 19.13% (PCR) in 920 porcine samples. Infection rates correlated with population density, GDP, and tourism. The study highlights the significant prevalence of *K. pneumoniae* in Chinese pigs and its implications for pig health and disease prevention.

Srivastava et al. reviewed Marburg virus disease comprehensively, discussing its pathophysiology, transmission routes, and the need for effective treatments and vaccines. Their review included a detailed analysis of past outbreaks, virus structure, and the current state of clinical management and research.

Melgarejo et al. developed an experimental model of tuberculosis in goats to evaluate new vaccines and treatments. Their study compared intranasal and endobronchial routes of infection, providing insights into the pathogenesis and immune response in goats. It demonstrated the importance of route-specific inoculations to replicate natural infections. Xue et al. investigated the presence of zoonotic pathogens *Anaplasma, Bartonella*, and *Rickettsia*, in Daurian ground squirrels in Hebei Province, China. The study highlights the genetic diversity of these bacteria in ground squirrels and notes that six identified species are pathogenic to humans, posing a potential health risk to local populations, particularly herders who are in close contact with these animals.

Ulloque-Badaracco et al. performed a meta-analysis on the seroprevalence of human toxocariasis in Latin America and the Caribbean, offering valuable data for epidemiological surveillance and prevention strategies. Their analysis included over 31,000 participants, identifying key risk factors and providing a comprehensive overview of the disease's prevalence in the region. Salamandane et al. assessed the genetic patterns of *Giardia duodenalis* and *Enterocytozoon bieneusi* in vegetables and fruits in Mozambique, highlighting their public health importance and zoonotic transmission potential. They used nested-PCR methods to detect these pathogens, identifying multiple novel sequences with high zoonotic transmission potential. Meng Y. et al. reviewed the complexities of diagnosing and treating bone cystic echinococcosis, underlining the need for improved diagnostic and therapeutic strategies. Their review covered imaging techniques, serological tests, and treatment options, highlighting the challenges in managing this complex disease.

Song et al. explored the antibacterial and anti-inflammatory effects of epigallocatechin gallate (EGCG) in treating canine periodontal disease, suggesting its potential as a therapeutic agent. They conducted both *in vitro* and *in vivo* experiments, showing that EGCG significantly reduced bacterial load and inflammation in a mouse model of periodontal disease. Lu et al. conducted a meta-analysis of the clinical characteristics of patients who died from hemorrhagic fever with renal syndrome (HFRS). They analyzed data from 37 articles involving 140,295 patients. The study found that those who died were typically older, more likely to smoke, and had hypertension and diabetes. These patients also exhibited severe symptoms like multiple organ dysfunction, shock, cerebral complications, heart and liver damage, and acute kidney injury. The findings highlight high-risk factors for mortality, aiding in clinical assessment and prognostication. Zhang Z. et al. examined the clinical characteristics and outcomes of acute kidney injury in patients with severe fever with thrombocytopenia syndrome (SFTS), identifying risk factors for poor prognosis. Their study provided detailed clinical data, highlighting the need for early identification and management of AKI in SFTS patients.

In conclusion, significant advances have been made in understanding zoonotic diseases through multi-omics and host-pathogen interaction studies. The research presented here emphasizes the importance of comprehensive surveillance, innovative diagnostic methods, and targeted prevention strategies. Several studies have revealed new insights into the genetic diversity, transmission dynamics, and ecological impacts of zoonotic pathogens. These findings underscore the necessity for larger-scale, interdisciplinary studies to fully understand the complexities of zoonotic diseases and their impact on public health. Future research should focus on integrating data from diverse ecosystems and host species within the One Health framework to develop more effective strategies for controlling zoonotic diseases.

